# Do Emotional Components of Alexithymia Mediate the Interplay between Cyberbullying Victimization and Perpetration?

**DOI:** 10.3390/ijerph14121530

**Published:** 2017-12-08

**Authors:** Sebastian Wachs, Ludwig Bilz, Saskia M. Fischer, Michelle F. Wright

**Affiliations:** 1 Department of Educational Studies, University of Potsdam, 14476 Potsdam, Germany; 2 Department of Health Sciences, Brandenburg University of Technology Cottbus-Senftenberg, 03046 Cottbus, Germany; ludwig.bilz@b-tu.de (L.B.); saskia.fischer@b-tu.de (S.M.F.); 3 Department of Psychology, Pennsylvania State University, PA 16802, USA; mfw5215@psu.edu; 4 Faculty of Social Studies, Masaryk University, 60200 Brno, Czechia

**Keywords:** alexithymia, cyberbully-victims, cyberbullying, cybervictimization, mediation

## Abstract

A substantial amount of research has revealed that cyberbully-victims have more emotional and behavioral problems than either cyberbullying victims or perpetrators. However, until now, little research has been conducted into the factors that contribute to the interplay between cyberbullying victimization and perpetration. The purpose of this study was to examine the relationship between cyberbullying victimization, perpetration, and two emotional components of alexithymia, namely difficulties in identifying and describing one’s own feelings. Self-report questions were administered to 1549 adolescents between 12 and 18 years old (*M* = 14.51; *SD* = 1.68; 42.1% (*n* = 652) male) from Germany and Thailand. Results showed that cyberbullying victimization and alexithymia are associated with cyberbullying perpetration. Moreover, alexithymia mediated the associations between cyberbullying victimization and adolescents’ cyberbullying perpetration. Consequently, we suggest that the ability to describe and identify one’s own feelings might be important for understanding the link between cyberbullying, victimization, and perpetration. The results may help develop prevention and intervention programs focused on reducing cyberbullying.

## 1. Introduction

Cyberbullying is a form of aggression that is intentional and repetitive, is based on an imbalance of power in favor of the perpetrator, and is carried out via information and communication technologies [1]. In the early years of bullying research, Olweus [2] stated that some young people are not only victims or only perpetrators, but are both simultaneously—so-called bully-victims. The same has been observed for cyberbullying [3,4,5]. In general, two different pathways for cyberbully-victims are possible: A cyberbully-to-cybervictim pathway in which a cyberbully becomes a cybervictim and a cybervictim-to-cyberbully pathway in which a cybervictim becomes a cyberbully. Since initial research on traditional bullying based on longitudinal data indicated that the victim-to-bully pathway is more common than the bully-to-victim pathway [6,7], we will focus on the cybervictim-to-cyberbully pathway in the present study.

There are several possible explanations for why a cybervictim might become a cyberbully. Some cyberbullying victims may engage in reactive aggression, intending to get revenge on cyberbullying perpetrators, while other cyberbullying victims may begin to cyberbully others because they feel frustrated and let down by their social environment. Other explanations may take into account observational learning, inappropriate coping strategies, and dynamic group processes [8]. Although there is a substantial body of literature indicating that bully-victims display the most psychological and behavioral difficulties compared with bullies or victims in both traditional and cyber bullying [9,10,11], little attention has been given to understanding why some cyberbullying victims become cyberbullying perpetrators. Cyberbullying victimization and perpetration might be directly related, although these variables may also be associated through the mediation of other variables. To reduce the risk of emotional and social problems associated with cyberbully-victim status, it is important to understand which factors contribute to becoming a cyberbully-victim. Alexithymia, in particular, might be one variable that might mediate the relationship between cyberbullying victimization and perpetration, but to date it has garnered little research attention.

The term alexithymia was introduced by Sifneos [12] and includes emotional components such as difficulties in identifying, describing, and interpreting one’s emotions and cognitive components such as externally-oriented thinking. Alexithymia can be considered a personal trait (primary alexithymia), or a state reaction evoked by stressful situations and traumatic experiences (secondary alexithymia) [13,14,15,16]. A substantial amount of research has revealed that alexithymic symptoms in children and adolescents can occur as a reaction evoked by stressful situations, traumatic experiences, and low social support [17]. Victims of cyberbullying experience extreme and chronic stress and they often lack social support, which is why they may be prone to developing alexithymia. In cyberbullying victims, alexithymia might also be seen as an unfavorable coping mechanism used to suppress and reject uncomfortable emotions that arise from cyberbullying victimization. Indeed, research reveals a relationship between both traditional and cyber bullying victimization and alexithymia [18,19,20].

Alexithymia, however, may also be associated with cyberbullying perpetration. According to the General Aggression Model [21], personality traits contribute to aggressive behaviors in humans. By the same token, a number of studies have revealed that individuals with alexithymia exhibit more interpersonal problems and that they are more likely to exhibit uncontrolled emotional responses [22,23,24]. Additionally, other studies have found that alexithymia can facilitate expressions of anger and aggressive behavior more generally [23] and that it is negatively correlated with the ability to cope with stress [25]. Furthermore, core characteristics of alexithymia include a lack of social attachment and social skills, which might further contribute to the association between alexithymia and cyberbullying perpetration [13,17]. Indeed, initial research has shown that both traditional and cyber bullies show higher levels of alexithymia [20,26].

Theoretically, alexithymia might be a mediator between cyberbullying victimization and perpetration, because the inability to modulate emotions through cognitive processes, as well as impairments in emotional awareness, can increase one’s engagement in impulsive actions and expressions of anger [24]. Cyberbullying others might be one expression of impulsive actions carried out in order to compensate for emotional deficits and anger in some cyberbullying victims.

Most studies on cyberbullying have been conducted in Western countries. Only a few studies have investigated cyberbullying among adolescents in non-Western samples such as Southeast Asia [27]. In order to enhance our knowledge about cyberbullying behavior among Thai adolescents and to understand what the cultural or individual determinants of adolescents’ cyberbullying behavior are, more cross-cultural research is needed that includes Western and non-Western participants. Including Western and non-Western participants in one study sample might also increase the informative value and therefore might help to develop prevention and intervention programs that show inter-cultural validity [11].

To summarize, a number of studies have shown that alexithymia is related to victimization, while others have found an association between alexithymia and perpetration in traditional and cyber bullying. However, no studies, to our knowledge, have thus far examined whether alexithymia mediates the associations between cyberbullying victimization and perpetration. The present study thus addresses this gap in the research. Achieving a better understanding of the role of alexithymia in the cybervictim-bully pathway in cyberbullying can help to further develop prevention and intervention programs designed to reduce cyberbullying. Thus, the study’s aim was to research the direct and indirect associations between cyberbullying victimization and perpetration, mediated by alexithymia. It was hypothesized that:

**Hypothesis** **1** **(H1).** As cyberbullying victimization increased, cyberbullying perpetration would increase.

**Hypothesis** **2** **(H2).** As cyberbullying victimization increased, alexithymia would increase.

**Hypothesis** **3** **(H3).** As alexithymia increased, cyberbullying perpetration would increase.

**Hypothesis** **4** **(H4).** The relationship between cyberbullying victimization and perpetration would be mediated by alexithymia, whereby cyberbullying victimization increased alexithymia, which in turn increased cyberbullying perpetration.

## 2. Materials and Methods

### 2.1. Participants

A total of 1549 adolescents between 12 and 18 years old (*M* = 14.51; *SD* = 1.68; 42.1% (*n* = 652) male) participated in this study. Adolescents were distributed in the following way across the countries: 54.8% (*n* = 849; *M* = 14.13; *SD* = 1.23, 50.3% (*n* = 427) male) were from Germany, and 45.2% (*n* = 700; *M* = 15; *SD* = 1.91, 32.1% (*n* = 225) male) were from Thailand. Overall, 34 participants were excluded from the analysis due to illogical responses, extremely one-sided response patterns, consistently filling in the extremes or with many questions unanswered. This corresponds to 2.19% of all participants. The decision to withdraw these data is supported by Tabachnick and Fidell [28] who state that if missing data represent less than 5% of the total sample deletion of the data is an acceptable loss.

There was a significant effect of age for country of origin, *t*(1544) = 10.51, *p* < 0.001, with Thai participants being older than German participants. Regarding sex differences by country of origin, a chi-square test revealed that the percentages of participants being male differed by country of origin significantly, χ^2^(1, *N* = 1549) = 51.86, *p* < 0.001. These differences in age and sex distributions among the two samples warrant the inclusion of age, sex, and country of origin as control variables in all further analyses.

### 2.2. Procedure

Ten public schools in Germany and five public schools in Thailand were informed of the study through invitation letters sent via email. This recruitment method resulted in three secondary schools from Germany, and one school from Thailand agreeing to take part in the research. Meetings were then arranged with interested schools to inform them about how they could participate. After the meeting, teachers distributed information letters to students and parents. Parents of adolescents under 18 years of age signed a written consent form to allow their children to participate in the research.

The data collection method used in Germany was an online survey, and in Thailand a paper-pencil format was used. Data were collected during regular school hours and trained research assistants were present during data collection to answer questions and ensure that adolescents filled the surveys out independently. Before completing the surveys, adolescents were informed that their participation in the study was anonymous, optional, that they could skip questions, and that they could stop participating at any time without giving a reason or fearing negative consequences. The research instruments were available in the German language. For the survey in Thailand, the research instruments were translated into Thai, and then a bilingual person who had not previously seen the original items reviewed and back-translated the research instruments.

### 2.3. Measures

#### 2.3.1. Cyberbullying Victimization and Perpetration

The Mobbing Questionnaire for Students by Jäger, Fischer, and Riebel [29] was used to measure cyberbullying victimization and cyberbullying perpetration with four items used for each subscale. The questionnaire includes a definition of cyberbullying that comprises the three central characteristics proposed by Olweus [2] (imbalance of power, repetition of the acts, and intention to hurt) and the use of ICT. For cyberbullying victimization, participants were asked “How many times has someone sent you threats, defamations, or other aggravating messages via the Internet/cell phone in the last twelve months?”, “… did someone spread rumors or defamations about you via the internet/cell phone…?”, “… did someone hand on private emails, chat messages, or pictures of you to others with the intention of exposing you…?”, “…did someone exclude you from a group in chats or online games…?”. The questions were repeated for cyberbullying perpetration. All items were responded to using a five-point ordinal scale, with response options being “Never” (=1), “Once or twice”, “Two or three times a month”, “About once a week” or “Several times a week” (=5). Both scales were created by taking the mean scores. Cronbach’s alphas of the cyberbullying victimization scale were also acceptable for the total sample (*α* = 0.72), and the German (*α* = 0.73) and Thai subsamples (*α* = 0.72). The results obtained in the confirmatory factor analysis (CFA) revealed a good fit of the cyberbullying victimization scale: *CFI* = 0.995; *TLI* = 0.984; *SRMR* = 0.017; *RMSEA* = 0.046; 90% of the confidence interval of the *RMSEA* = 0.018–0.080. Cronbach’s alphas of the cyberbullying perpetration scale were acceptable for the total sample (*α* = 0.76), and the German (*α* = 0.79) and Thai subsamples (*α* = 0.71). The results obtained in the CFA revealed a good fit of the cyberbullying perpetration scale: *CFI* = 0.999; *TLI* = 0.996; *SRMR* = 0.008; *RMSEA* = 0.024; 90% of the confidence interval of the *RMSEA* = 0.001–0.061.

#### 2.3.2. Emotional Components of Alexithymia

Emotional components of alexithymia were measured by two subscales of the Toronto Alexithymia Scale [30,31], namely (1) difficulties identifying one’s own feelings (DIF) (seven items) and (2) difficulties in describing one’s own feelings (DDF) (five items). As recommended by previous research, we combined these subscales into one sum scored scale [32,33,34,35,36]. Since the third subscale, externally-oriented thinking (EOT), showed low reliability in previous research and previous research revealed that alexithymia can be reliable measured among adolescents without using the EOT subscale [32,36] we did not measure this subscale in the current study. To measure DIF and DDF, participants were asked to rate to what extent they agreed with statements such as “It is difficult for me to reveal my innermost feelings, even to close friends” or “It is difficult for me to find the right words for my feelings” on a five-point Likert scale, ranging from “strongly disagree” (=1) to “strongly agree” (=5). The higher participants scored on this scale the higher their alexithymia; Cronbach’s alphas were good for the total sample (*α* = 0.89), and the German (*α* = 0.88) and Thai subsamples (*α* = 0.89).

A CFA revealed that the item “I’m able to describe my feelings easily” had standardized factor loadings below the recommended 0.32 threshold [37]. Therefore, this item was removed which resulted in a good fit of the alexithymia scale: *CFI* = 0.964; *TLI* = 0.952; *SRMR* = 0.029; *RMSEA* = 0.070; 90% of the confidence interval of the *RMSEA* = 0.062–0.078 and a higher Cronbach’s alpha for the 11-item scale (*α* = 0.91).

#### 2.3.3. Demographic Variables

Socio-demographics were measured by asking for information on students’ age (years) and sex (male; female).

### 2.4. Data Analyses

A *t*-test was used to investigate bivariate country of origin, age, and sex differences in cyberbullying victimization, perpetration, and alexithymia with Cohen’s *d* to measure the effect size. Confirmatory Factor Analyses (CFA) were completed in Mplus 7.4 software (Los Angeles, CA, USA) [38]. The fit of the model and CFA were examined by taking into account the following indices: the Root Mean Square Error of Approximation (RMSEA), the Standardized Root Mean Square Residual (SRMR), the Comparative Fit Index (CFI), and Tucker-Lewis Index (TLI). RMSEA and SRMR values of 0.05 or less and CFI and TLI values of 0.95 or greater are interpreted as evidence of models that fit well [39].

The PROCESS macro was used to conduct regression-based statistical mediation in order to analyze whether cyberbullying victimization (IV) predicted a higher likelihood of cyberbullying perpetration (DV) via alexithymia (M), while controlling for age, sex, and country of origin. The significance of the indirect effect was estimated using 1000 bootstrapped resamples [40]. As an effect size, Cohen’s *f*^2^ was used [41], *f*^2^ ≥ 0.10, *f*^2^ ≥ 0.25, and *f*^2^ ≥ 0.40 represent small, medium, and large effect sizes, respectively. Post hoc power analysis showed that the sample size of *n* = 1549 is large enough to reveal even very small effects of *f*^2^ = 0.01 with a power of 0.88 in the planned regression analyses with five predictors: country of origin, age, sex, alexithymia, and cyberbullying victimization, and an alpha of 0.05 using G*Power 3 to calculate the post hoc power analysis [42].

Before we conducted the mediation analysis, we tested the following assumptions of linear regression: multicollinearity, outliers, and the assumption of normality. The examination of the Tolerance Index and Variance Inflation Factor (VIF) indicated that multicollinearity was not an issue among our independent variables (VIFs < 2.0). Univariate outliers were winsorized by replacing values beyond the 5th and 95th percentile by exactly these values. Inspection of the P–P-plot and histogram revealed that the assumption of normality was violated. However, due to the large sample size, we assume that assumptions of regression were sufficiently met.

## 3. Results

### 3.1. Descriptive Results

Independent-sample *t*-tests were conducted to compare mean levels of cyberbullying victimization, perpetration, and alexithymia by country of origin (see Table 1). The analyses revealed that country of origin did not have an effect on cyberbullying victimization. However, Thai participants reported more frequently cyberbullying perpetration and higher levels of alexithymia compared with German participants. Mean level comparison of cyberbullying victimization, perpetration, and alexithymia by sex were conducted by independent-samples *t*-tests (see Table 1). The results showed that boys were significantly more likely to report cyberbullying victimization compared with girls. Furthermore, girls were more likely to report cyberbullying perpetration and alexithymia compared with boys.

Regarding age, bivariate correlation analyses showed that age and cyberbullying victimization were not significantly correlated, *r*(1547) = 0.039, *p* = 0.128. However, with increasing age cyberbullying perpetration significantly increased, *r*(1544) = 0.221, *p* < 0.001. Also, age and alexithymia were significantly positively correlated, *r*(1544) = 0.197, *p* < 0.001, indicating that with increasing age, alexithymia increased.

### 3.2. Direct and Indirect Associations between Cyberbullying Victimization and Perpetration, Mediated by Alexithymia

To answer the research question of whether the association between cyberbullying victimization and cyberbullying perpetration was mediated by alexithymia, we conducted a regression-based mediation analysis with cyberbullying victimization as the independent variable, alexithymia as a mediator, and cyberbullying perpetration as the outcome, while controlling for age, sex, and country of origin. Both the regression model to predict the mediator (alexithymia), *F*(4, 1541) = 78.515, *p* < 0.001, and the model to predict the outcome (cyberbullying perpetration), *F*(5, 1541) = 61.186, *p* < 0.001, were significant (see Table 2). Approximately 17% of the variance in alexithymia was accounted for by cyberbullying victimization, country of origin, age, and sex (*R*^2^ = 0.170), indicating a large effect (Cohen’s *f*^2^ = 0.496). Approximately 16.6% of the variance in cyberbullying perpetration was accounted for by cyberbullying victimization, alexithymia, age, sex, and country of origin (*R*^2^ = 0.166), indicating a large effect (Cohen’s *f*^2^ = 0.488).

**Figure 1:**
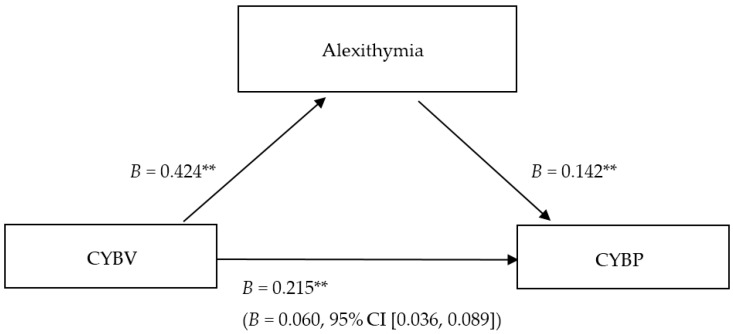
Direct and indirect effects of cyberbullying victimization (CYBV) and alexithymia on cyberbullying perpetration (CYBP). The indirect effect of cyberbullying victimization on cyberbullying perpetration via alexithymia is reported in parentheses with bootstrapped CI. ** *p* < 0.001.

The mediation analysis revealed a positive direct effect of cyberbullying victimization on alexithymia (*B* = 0.424; *p* < 0.001, 95% CI (0.363, 0.485)) and a positive direct effect of cyberbullying victimization on cyberbullying perpetration (*B* = 0.215; *p* < 0.001, 95% CI (0.156, 0.274)). Higher alexithymia increased the likelihood of higher cyberbullying perpetration (*B* = 0.142, *p* < 0.001, 95% CI (0.097, 0.188)). As Figure 1 shows, the mediation analysis confirmed alexithymia as a partial mediator of the relationship between cyberbullying victimization and perpetration. The positive indirect effect of cyberbullying victimization on cyberbullying perpetration through alexithymia was significant (*B* = 0.060, 95% CI (0.036, 0.089)).

In order to investigate whether the observed mediation model is moderated by age, sex, or country of origin, moderated mediation analyses were conducted using PROCESS Model 59. The results showed no moderation effect on the mediation model. More information can be requested from the first author. Due to the cross-sectional data, we also investigated in another mediation model whether cyberbullying perpetration (IV) predicted higher likelihood of cyberbullying victimization (DV) via alexithymia (M), while controlling for country of origin, age, and sex. The results showed a positive direct effect (*B* = 0.151, *p* < 0.001, 95% CI (0.110, 0.193)) and a positive indirect effect (*B* = 0.055, 95% CI (0.033, 0.083)). More information can be requested from the first author.

## 4. Discussion

Cyberbully-victims have shown to be the most problematic cyberbullying group when compared with cyberbullying perpetrators and cyberbullying victims [5,9,10,11]. However, until now, there has been little research that has attempted to understand whether the interplay between cyberbullying victimization and perpetration might be mediated through other factors. Thus, the purpose of the present study is to examine whether the associations between cyberbullying victimization and perpetration are mediated through emotional components of alexithymia in a sample of 1549 adolescents aged between 12 and 18 from Germany and Thailand.

We found empirical support for our first hypothesis, that cyberbullying victimization and perpetration were positively correlated which is in line with previous research, showing an overlap between these groups in the context of both traditional and cyber bullying [3,4,5,11]. This association might be explained by reactive aggression, observational learning, inappropriate coping strategies, and dynamic group processes [8]. To date, there exists a paucity of research that compares the transition into bullying from victimization across both cyber and traditional settings. However, it has been shown that the number of bully-victims in cyber settings far exceeds the one found in traditional settings [3,5,11]. This difference might be explained by the lack of face-to-face contact in online communication and interaction which may limit the ability to empathize for others and promote the release of inhibition.

In accordance with our second hypothesis, the analysis revealed that cyberbullying victimization was positively associated with alexithymia. This might be explained by the likelihood that cyberbullying victims experience extreme and chronic stress and often lack social support, potentially making them more likely to develop alexithymia. Previous research has shown that extreme stress can lead to alexithymia in adolescents [13,14,15,16,17] and this is also in line with past traditional and cyber bullying research [18,19,20]. In cyberbullying victims, alexithymia may also work as a dysfunctional coping mechanism to avoid exploring one’s stressful feelings. It is worth mentioning that alexithymia as a personal trait (primary alexithymia) might also be a risk factor for cyberbullying victimization and not—as assumed in this study—a consequence, as a state reaction evoked by stressful situations and traumatic experiences (secondary alexithymia).

The third hypothesis had support, where alexithymia was positively correlated with cyberbullying perpetration. This finding is in line with previous work [20,26] and theoretically with the General Aggression Model [21] that postulates that personality traits contribute to aggressive behaviors in humans. Among some adolescents, alexithymia facilitates interpersonal problems, uncontrolled emotional responses, expressions of anger, and aggressive behavior, which has been broadly shown in other studies on the associations between alexithymia and aggressive behavior [22,23,24,25]. This might put alexithymic adolescents at risk for engagement in cyberbullying as perpetrator.

Findings concerning the fourth hypothesis extend the literature by revealing evidence that: the relationship between cyberbullying victimization and perpetration is mediated by alexithymia; and cyberbullying victimization increases alexithymia, which in turn increases cyberbullying perpetration. This mediated association might be explained by the inability to modulate emotions through cognitive processes, as well as impairments in emotional awareness which can increase one’s engagement in impulsive actions and expressions of anger. The associations between cyberbullying victimization, perpetration, and alexithymia might also partially explain why some cyberbully-victims tend to show more social and psychological difficulties compared with pure bullies or victims because research has shown that alexithymia predicts mental disorders and difficulties in social relationships [43].

Since the observed indirect effect was small, more research is needed to identify other mediators in the interplay between cyberbullying victimization and perpetration. This might be important in further advancing our understanding of why cyberbullying victims become cyberbullying perpetrators and how we might be able to prevent this transition. Future follow-up analysis would also benefit from a wider range of control variables (i.e., socioeconomic status, living in rural areas) that have been shown to influence alexithymic features in adolescents [17].

Finally, an interesting result from the present study is that girls reported higher levels of alexithymia. This finding contrasts with Levant’s [44] “Normative Male Alexithymia” hypothesis that postulates that men show higher alexithymia as a result of society’s traditional idea of masculinity. Studies on gender and alexithymia among adolescents and young adults showed contrary results. For example, in one study with Finish adolescents between 13 and 18 years, girls reported more alexithymia than boys [43]. In addition, considering the emotional and cognitive subscales of the TAS-20 separately can lead to varying gender differences. Accordingly, some research has found that female participants scored higher on the DIF subscale, while male participants displayed higher levels on the EOT subscale [45,46]. In the present study, EOT was not measured which might explain the gender differences in favor of girls. Yet, other studies did not find any gender differences among male and female participants on alexithymia [47]. Gender differences might also be influenced by the categorization strategy (using a specific cut-off value) of alexithymic participants. In one study, girls were more likely to be classified as alexithymic under a categorical approach and boys were more likely under a continuous approach [48]. Clearly, more research on the gender differences of alexithymia among adolescents is needed.

## 5. Limitations and Strengths

Some limitations of the present research need to be mentioned. First, due to the cross-sectional study design, caution must be used when establishing causal relationships between cyberbullying victimization, perpetration, and alexithymia. It might also be possible that cyberbullying perpetration leads to cyberbullying victimization and alexithymia appears to be a risk factor of cyberbullying victimization and not a consequence. Longitudinal studies would help to identify the temporal ordering of the study variables. Second, the sample cannot be considered representative. Therefore, caution is recommended in using the results to make generalizations. In addition, another limitation related to the sample is that there are far more girls than boys in the Thai sample (32.1% vs. 67.9%) which might have influenced the bivariate analyses presented in Table 1. Thus, these bivariate findings should be interpreted with some caution. In the mediation analyses, country of origin and sex were included as control variables. Therefore, it can be assumed that these sample characteristics did not severely influence the results of the mediation analyses. Third, all of the data relies exclusively on self-reports. Thus, the correlates might be inflated through shared method variance. Finally, we only assessed emotional facets of alexithymia, namely difficulties in identifying and describing one’s own emotion. Further research is needed to clarify whether externally-oriented thinking also mediates the interplay between cyberbullying victimization and perpetration.

Nevertheless, this study also exhibits a number of strengths and extends the body of research in several ways. First, the sample of this study was large, which made it possible to investigate cyberbullying behaviors among adolescents. Second, the present study is one of the first to identify the mediating role of alexithymia in the interplay between cyberbullying victimization and perpetration, and therefore enhances our understanding of factors that might explain why cyberbullying victims become cyberbullying perpetrators. Second, through the inclusion of Thai adolescents in the study sample, the present study extends our knowledge of cyberbullying in non-Western societies. Third, the research instruments employed in this study have been standardized and previously validated. In addition, to improve the validity of responses, the participants were given a definition of cyberbullying before answering the questions about cyberbullying.

## 6. Practical Implications

We recommend the development of educational programs about the negative emotional effects of cyberbullying victimization, as this study shows that cyberbullying victimization poses risks to students’ well-being and emotional development. Additionally, identifying and describing one’s own emotions appears to be an important issue in helping cyberbullying perpetrators to overcome their aggressive behavior. Educating adolescents on how to describe their own emotions might stop them from becoming cyberbully-victims and may prevent further negative outcomes associated with the cyberbully-victim status. One promising approach may be to include emotional skills training in cyberbullying intervention and prevention programs. These measures might include, among others, teaching the necessity and worth of emotions, educating students in how to understand one’s own emotions, and teaching vocabulary and ways to express positive and negative emotions. Most current intervention and prevention programs against cyberbullying continue to focus on understanding the emotions of others (empathy). However, the present study shows that it may be crucial to understand and express one’s own emotions too. The findings also point out the need for rapid intervention in cyberbullying and the emotional support of cyberbullying victims, which can decrease the likelihood that cyberbullying victims will process experiences dysfunctionally through alexithymia and consequently become less likely to become cyberbullying perpetrators.

## 7. Conclusions

The present study further advances our understanding of the direct and indirect associations between cyberbullying victimization, perpetration, and alexithymia in adolescents. The current study provides evidence that the effects of cyberbullying victimization on cyberbullying perpetration are partially mediated by alexithymia. Thus, alexithymia appears to be key to understanding the interplay between cyberbullying victimization and perpetration. Intervention and prevention programs against cyberbullying must consider the negative emotional effects of cyberbullying victimization on adolescents’ emotional development and educating adolescents about the importance of the ability to describe their own emotions. Prevention and intervention programs against cyberbullying should include emotional skills training (i.e., teaching vocabulary and ways to express positive and negative feelings) to prevent cyberbullying victims from becoming cyberbullying perpetrators.

## Figures and Tables

**Table 1:** Bivariate differences in the study variables by sex and country of origin.

Variables	Country of Origin				*t*-Test			Sex					*t*-Test	
	German		Thai					Female		Male				
	*M*	*SD*	*M*	*SD*	*t*	*p*	*d*	*M*	*SD*	*M*	*SD*	*t*	*p*	*d*
Cyberbullying Victimization	1.20	0.54	1.24	0.56	1.41	0.156	-	1.17	0.45	1.28	0.66	3.77	0.001	0.194
Cyberbullying Perpetration	2.15	0.64	2.48	0.63	10.62	0.001	0.519	2.36	0.63	2.20	0.64	4.72	0.001	0.251
Alexithymia	26.06	10.08	32.35	9.78	12.40	0.001	0.633	31.09	10.09	25.90	10.08	9.96	0.001	0.514

**Table 2:** Mediation analysis of cyberbullying victimization on perpetration via alexithymia.

Predictor	Outcome	*B*	95% CI	
			*LL*	*UL*
*Constant*		0.417 **	0.116	0.719
Cyberbullying victimization	Alexithymia	0.424 **	0.363	0.485
Country of origin ^Thai^	Alexithymia	−0.208 **	−0.278	−0.138
Age	Alexithymia	0.099 **	0.079	0.120
Sex ^Female^	Alexithymia	−0.107 **	−0.176	−0.038
*R*^2^		0.170		
*F*		78.515 **		
*Constant*		0.948 *	0.674	1.221
Cyberbullying victimization	Cyberbullying perpetration	0.215 **	0.156	0.274
Alexithymia	Cyberbullying perpetration	0.142 **	0.097	0.188
Country of origin ^Thai^	Cyberbullying perpetration	0.273 **	0.208	0.337
Age	Cyberbullying perpetration	0.047 **	0.028	0.066
Sex ^Female^	Cyberbullying perpetration	0.148 **	0.085	0.210
*R*^2^		0.166		
*F*		61.186 **		

Note: *N* = 1.218. *LL* = lower limit, *UL =* upper limit. * *p* < 0.01 ** *p* < 0.001.
